# Supplementary material for: The Small RNA Universe of Capitella teleta
Source: Front Mol Biosci. 2022 Feb 25;9:802814. doi: 10.3389/fmolb.2022.802814 (PMC8915122; doi:10.3389/fmolb.2022.802814)
Supplement: Supplementary file 1 [file DataSheet1.ZIP › Supplement/known/CAPTEscaffold_19_2482.pdf]

Provisional ID	: CAPTEscaffold_19_2482
Score total	: 43751.3
Score for star read(s)	: 3.9
Score for read counts	: 43741.6
Score for mfe	: 1.2
Score for randfold	: 1.6
Score for cons. seed	: 3
Total read count	: 85809
Mature read count	: 80446
Loop read count	: 0
Star read count	: 5363

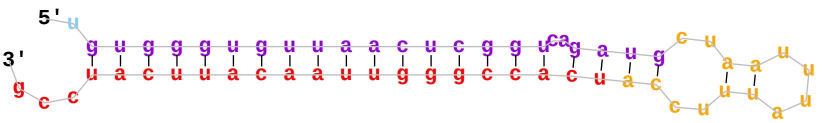

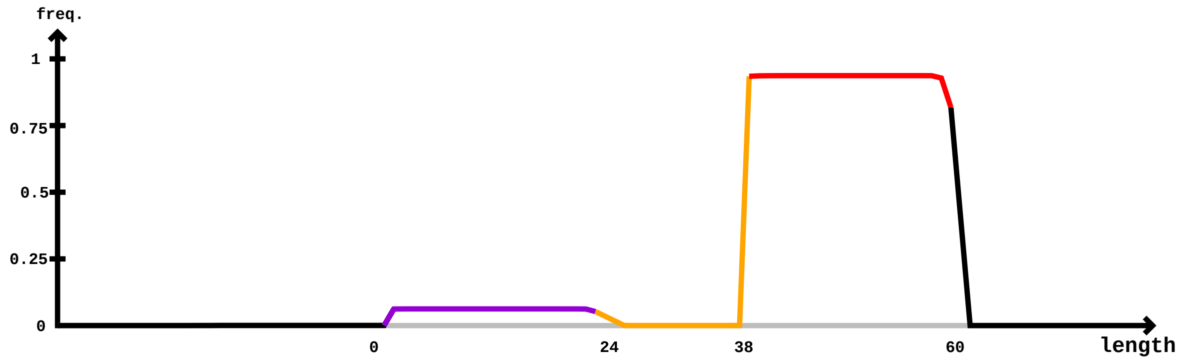

Star   Mature

```
5'- cugcggaacuugacacacuuaugucuacucccuguggguguuaacucggucagaugcuaauuuauuuccaucaccggguuaacauucauccgggaaggccauacaaaucuac   -3'   obs
    cugcggaacuugacacacuuaugucuacucccuguggguguuaacucggucagaugcuaauuuauuuccaucaccggguuaacauucauccgggaaggccauacaaaucuac         exp
    cugcggaacuugacacacuuaugucuacucccuguggguguuaacucggucagaugcuaauuuauuuccaucaccggguuaacauucauccgggaaggccauacaaaucuac         known
    ...................(((((((..((((.(((((((((((((((((..((((..((.....))..))))))))))))))))))))).))))))).)))).........       reads   mm   sample

    ............acacacuuaugucuacucccu...............................................................................       7       0    seq
    .............cacacuuaugucuacucccu...............................................................................       12      0    seq
    ..............acacuuaugucuacucccu...............................................................................       1       0    seq
    ...............cacuuaugucuacucccu...............................................................................       19      0    seq
    ................................uguggguguuaacucggucaga..........................................................       2       0    seq
    .................................guggguguuaacucgguc.............................................................       4       0    seq
    .................................guggguguuaacucggCca............................................................       1       1    seq
    .................................guggguguuaacucgguca............................................................       5       0    seq
    .................................guggguguuaacucggucag...........................................................       27      0    seq
    .................................guggguguuaacAcggucaga..........................................................       2       1    seq
    .................................guggguguuaacucggucagG..........................................................       1       1    seq
    .................................guggguguuaacucggucaCa..........................................................       1       1    seq
    .................................guggguguuaacucAgucaga..........................................................       1       1    seq
    .................................gugggAguuaacucggucaga..........................................................       1       1    seq
    .................................guggguguuaacuUggucaga..........................................................       3       1    seq
    .................................gugggugu Caacucggucaga..........................................................       2       1    seq
    .................................guggguguuaacucUgucaga..........................................................       1       1    seq
    .................................gugggGguuaacucggucaga..........................................................       1       1    seq
    .................................guAgguguuaacucggucaga..........................................................       3       1    seq
    .................................guggguguuaacucggucagU..........................................................       1       1    seq
    .................................guggguguuGacucggucaga..........................................................       1       1    seq
    .................................guggguguuaacucggucaga..........................................................       799     0    seq
    .................................guggguguuaacuAggucagau.........................................................       1       1    seq
    .................................guggguguuaacucggucagau.........................................................       114     0    seq
    .................................guggguguuaacucggucagauA........................................................       5       1    seq
    .................................guggguguCaacucggucagaug........................................................       2       1    seq
    .................................guggguguuaacucggucagauU........................................................       8       1    seq
    .................................guggguguuaacucggucagaAg........................................................       2       1    seq
    .................................guggguguuaacAcggucagaug........................................................       1       1    seq
    .................................guggguguuaacucgguAagaug........................................................       2       1    seq
    .................................guggguguuaacucgguUagaug........................................................       2       1    seq
```

Star Mature

```
cugcggaacuugacacacuuaugucuacucccuguggguguuaacucggucagaugcuaauuuauuuccaucaccggguuaacauucauccgggaaggccauacaaaucuac
.................................guggguguuaacuAggucagaug........................................................ 1 1 seq
.................................guggguguuaacuUggucagaug........................................................ 18 1 seq
.................................guggguguuaacucAgucagaug........................................................ 11 1 seq
.................................gugggCguuaacucggucagaug........................................................ 1 1 seq
.................................guggguUuuaacucggucagaug........................................................ 1 1 seq
.................................guggguguAaacucggucagaug........................................................ 2 1 seq
.................................guggguguuaacucggucagUug........................................................ 1 1 seq
.................................guggguguuaacucgCucagaug........................................................ 1 1 seq
.................................guggguguuaacucggucagaug........................................................ 3852 0 seq
.................................gugggAguuaacucggucagaug........................................................ 1 1 seq
.................................guggguguuaacucggucUgaug........................................................ 1 1 seq
.................................Uuggguguuaacucggucagaug........................................................ 6 1 seq
.................................guggguguuaacucggucagGug........................................................ 1 1 seq
.................................gugAguguuaacucggucagaug........................................................ 9 1 seq
.................................gugUguguuaacucggucagaug........................................................ 1 1 seq
.................................guggguguuaacucgAucagaug........................................................ 1 1 seq
.................................guAgguguuaacucggucagaug........................................................ 24 1 seq
.................................gugggugAuaacucggucagaug........................................................ 1 1 seq
.................................guggguguuaacucggAcagaug........................................................ 2 1 seq
.................................gCggguguuaacucggucagaug........................................................ 1 1 seq
.................................guggguguuaaGucggucagaug........................................................ 1 1 seq
.................................guggguguuUacucggucagaug........................................................ 1 1 seq
.................................guggguguuaacucggucagaugc....................................................... 1 0 seq
.................................guggguguuaacucggucagaugA....................................................... 14 1 seq
.................................guggguguuaacucggucagauUc....................................................... 1 1 seq
.................................guggguguuaacucggucagaugU....................................................... 366 1 seq
.................................guggguguuaacucggucagaugUu...................................................... 13 1 seq
..................................uggguguuaacucggucaga.......................................................... 5 0 seq
..................................uggguguuaacucggucagau......................................................... 2 0 seq
..................................uggguguuaacucggucagaug........................................................ 17 0 seq
..................................uggguguuaacucggucagaugU....................................................... 5 1 seq
..................................uggguguuaacucggucagaugA....................................................... 1 1 seq
..................................uggguguuaacucggucagaugc....................................................... 3 0 seq
..................................uggguguuaacucggucagaugcu...................................................... 2 0 seq
...................................ggguguuaacucggucag........................................................... 1 0 seq
......................................uguuaacucggucagaug........................................................ 1 0 seq
.....................................................................Cucaccggguuaacauucaucc..................... 2 1 seq
.....................................................................aucaccggguuaacauucaucc..................... 4 0 seq
.....................................................................Cucaccggguuaacauucauccg.................... 1 1 seq
.....................................................................aucaccggguuaacauucaucUg.................... 1 1 seq
.....................................................................aucaccggguuaacauucauccg.................... 6 0 seq
......................................................................ucaccggguuaacauucau....................... 14 0 seq
......................................................................ucaccggCuuaacauucauc...................... 1 1 seq
......................................................................ucaccggguuaacauucauA...................... 2 1 seq
......................................................................ucaccggguuaacauucauU...................... 1 1 seq
......................................................................ucaccgAguuaacauucauc...................... 2 1 seq
......................................................................ucaccggguAaacauucauc...................... 1 1 seq
......................................................................ucaccggguuaacauAcauc...................... 3 1 seq
......................................................................ucaccggguuaacauucaAc...................... 1 1 seq
......................................................................Gcaccggguuaacauucauc...................... 2 1 seq
......................................................................ucaccggguuaUcauucauc...................... 1 1 seq
......................................................................ucacUggguuaacauucauc...................... 1 1 seq
......................................................................ucaccggguuaacauuUauc...................... 1 1 seq
......................................................................ucaccggguuaacauucauc...................... 730 0 seq
......................................................................ucaccggguuaacauucauG...................... 2 1 seq
......................................................................ucaccAgguuaacauucauc...................... 4 1 seq
......................................................................uUaccggguuaacauucauc...................... 1 1 seq
......................................................................ucaccgggCuaacauucauc...................... 1 1 seq
......................................................................ucaccggguuaacauuGauc...................... 1 1 seq
......................................................................Acaccggguuaacauucauc...................... 3 1 seq
......................................................................ucaccggUuuaacauucaucc..................... 2 1 seq
......................................................................ucaccggguuGacauucaucc..................... 1 1 seq
......................................................................ucaccggguuaacauucauAc..................... 22 1 seq
......................................................................ucaccgUguuaacauucaucc..................... 5 1 seq
......................................................................ucGccggguuaacauucaucc..................... 1 1 seq
......................................................................ucaccggguuaaUauucaucc..................... 2 1 seq
......................................................................ucaccggguuaacUuucaucc..................... 1 1 seq
......................................................................ucaccggguuaacauuUaucc..................... 14 1 seq
......................................................................ucaccgCguuaacauucaucc..................... 1 1 seq
......................................................................ucaccggguuaacauucaAcc..................... 4 1 seq
```

Star **Mature**

cugcggaacuugacacacuuaugucuacucccugugggguguuaacucggucagaugcuaauuuauuuccaucaccggguuaacauucauccgggaaggccauacaaaucuac			
....ucaccggguuaacauucaucc.....................	9218	0	seq
....ucaccggguuaacauucGucc.....................	5	1	seq
....ucaccggguuaaAauucaucc.....................	3	1	seq
....ucaccggguuaacauucaucA.....................	6	1	seq
....ucaccAgguuaacauucaucc.....................	26	1	seq
....ucaccggguuaacauucUucc.....................	1	1	seq
....ucaccggguuaacauucaCcc.....................	2	1	seq
....ucaccggguuaGcauucaucc.....................	2	1	seq
....Ncaccggguuaacauucaucc.....................	3	1	seq
....ucUccggguuaacauucaucc.....................	1	1	seq
....ucaccggguCaacauucaucc.....................	6	1	seq
....uNaccggguuaacauucaucc.....................	1	1	seq
....uUaccggguuaacauucaucc.....................	9	1	seq
....ucaccggguuaacauucaucU.....................	7	1	seq
....Gcaccggguuaacauucaucc.....................	16	1	seq
....ucaccggguAaacauucaucc.....................	10	1	seq
....ucacUggguuaacauucaucc.....................	8	1	seq
....ucaccCgguuaacauucaucc.....................	1	1	seq
....ucaccggCuuaacauucaucc.....................	1	1	seq
....ucaUcggguuaacauucaucc.....................	2	1	seq
....Acaccggguuaacauucaucc.....................	33	1	seq
....ucaccgAguuaacauucaucc.....................	13	1	seq
....ucaccggguuaaGauucaucc.....................	1	1	seq
....ucaccggguuaacauuAaucc.....................	4	1	seq
....ucaccggguuaacauAcaucc.....................	4	1	seq
....ucaccggguuaacaCucaucc.....................	1	1	seq
....ucacAggguuaacauucaucc.....................	6	1	seq
....ucaccggguuaUcauucaucc.....................	1	1	seq
....uAaccggguuaacauucaucc.....................	2	1	seq
....ucaccggguuaacaAucaucc.....................	5	1	seq
....ucaccgggGuaacauucaucc.....................	2	1	seq
....ucaccgggCuaacauucaucc.....................	2	1	seq
....ucaccggAuuaacauucaucc.....................	2	1	seq
....ucaccgggAuaacauucaucc.....................	4	1	seq
....ucaccggguuaacauuGauccg....................	3	1	seq
....ucaccggguuaacauucauccC....................	6	1	seq
....uGaccggguuaacauucauccg....................	5	1	seq
....ucaccggguuaCcauucauccg....................	2	1	seq
....ucaccgCguuaacauucauccg....................	5	1	seq
....ucaccggguuaaAauucauccg....................	19	1	seq
....ucaAcggguuaacauucauccg....................	7	1	seq
....Ccaccggguuaacauucauccg....................	7	1	seq
....ucaccgggAuaacauucauccg....................	26	1	seq
....ucaccggguuaacauCcauccg....................	10	1	seq
....ucaccggguuaacauuAauccg....................	23	1	seq
....ucaccggguCaacauucauccg....................	15	1	seq
....ucaccggguuUacauucauccg....................	4	1	seq
....uAaccggguuaacauucauccg....................	28	1	seq
....ucUccggguuaacauucauccg....................	6	1	seq
....ucaccggguuaacCuucauccg....................	1	1	seq
....ucaccgggGuaacauucauccg....................	7	1	seq
....ucaccggguuCacauucauccg....................	1	1	seq
....ucaccggguuaacauucaucGg....................	12	1	seq
....uNaccggguuaacauucauccg....................	2	1	seq
....ucaccggguuaacauucauccA....................	131	1	seq
....ucaccggguuaaUauucauccg....................	4	1	seq
....Ncaccggguuaacauucauccg....................	27	1	seq
....ucaccggguuaacauucaAccg....................	27	1	seq
....uUaccggguuaacauucauccg....................	61	1	seq
....ucaccggguuaacauGcauccg....................	2	1	seq
....ucaccgggCuaacauucauccg....................	14	1	seq
....ucaccggguuaacauucauUcg....................	12	1	seq
....ucaccggguuaacaAucauccg....................	22	1	seq
....ucaccggguuaacauuCuccg....................	3	1	seq
....ucaccggguGaacauucauccg....................	2	1	seq
....ucaccggguAaacauucauccg....................	53	1	seq
....ucaccggAuuaacauucauccg....................	3	1	seq
....ucaccggguuaacauucaucAg....................	58	1	seq
....ucaccggguuaacauucauAcg....................	121	1	seq
....ucaccggguuaacauucaCccg....................	10	1	seq

Star **Mature**

```
cugcggaacuugacacacuuaugucuacucccugugggguguuaacucggucagaugcuaauuuauuuccaucaccggguuaacauucauccgggaaggccauacaaaucuac
.......................................................................ucaccggguuaacauucaucUg....................	202	1	seq
.......................................................................ucaccCgguuaacauucauccg....................	4	1	seq
.......................................................................ucaccggUuuaacauucauccg....................	13	1	seq
.......................................................................ucaccAgguuaacauucauccg....................	229	1	seq
.......................................................................ucaGcggguuaacauucauccg....................	5	1	seq
.......................................................................ucaccggguuaacaCucauccg....................	10	1	seq
.......................................................................ucCccggguuaacauucauccg....................	6	1	seq
.......................................................................ucacAggguuaacauucauccg....................	29	1	seq
.......................................................................ucaccggguuaacauucUuccg....................	3	1	seq
.......................................................................ucacUggguuaacauucauccg....................	63	1	seq
.......................................................................ucGccggguuaacauucauccg....................	13	1	seq
.......................................................................ucaccggguuaacauucaGccg....................	6	1	seq
.......................................................................ucaccggCuuaacauucauccg....................	4	1	seq
.......................................................................ucaccggguuaacGuucauccg....................	10	1	seq
.......................................................................Gcaccggguuaacauucauccg....................	86	1	seq
.......................................................................ucaccggguuaacaGucauccg....................	6	1	seq
.......................................................................ucaccggguuaacauucauccU....................	78	1	seq
.......................................................................ucaccggguuaUcauucauccg....................	9	1	seq
.......................................................................ucaccggguuaGcauucauccg....................	9	1	seq
.......................................................................ucaccggguuaacauAcauccg....................	57	1	seq
.......................................................................ucaccggguuaacauucGuccg....................	20	1	seq
.......................................................................ucaccgAguuaacauucauccg....................	76	1	seq
.......................................................................Acaccggguuaacauucauccg....................	256	1	seq
.......................................................................ucaccggguuaaGauucauccg....................	8	1	seq
.......................................................................ucaccggguuaacUuucauccg....................	9	1	seq
.......................................................................ucaccggguuaacauucauGcg....................	4	1	seq
.......................................................................ucaccUgguuaacauucauccg....................	6	1	seq
.......................................................................ucacGggguuaacauucauccg....................	1	1	seq
.......................................................................ucaccggguuaacauuUauccg....................	35	1	seq
.......................................................................ucaUcggguuaacauucauccg....................	11	1	seq
.......................................................................ucaccggguuGacauucauccg....................	28	1	seq
.......................................................................ucaccggguuaacauucauccg....................	66996	0	seq
.......................................................................ucaccgUguuaacauucauccg....................	16	1	seq
.......................................................................ucaccggguuaacauucauccgC...................	4	1	seq
.......................................................................ucaccggguuaacauucauccgg...................	3	0	seq
.......................................................................ucaccggguuaacauucauccgU...................	874	1	seq
.......................................................................ucaccggguuaacauucauccgA...................	60	1	seq
........................................................................caccggguuaacauuAaucc.....................	1	1	seq
........................................................................caccggguuaacauucaucc.....................	4	0	seq
........................................................................caccggguuaacauucaAccg....................	2	1	seq
........................................................................caccggguuaaAauucauccg....................	3	1	seq
........................................................................caccggguuaacauucauAcg....................	1	1	seq
........................................................................caccggguuaacaAucauccg....................	1	1	seq
........................................................................Uaccggguuaacauucauccg....................	2	1	seq
........................................................................caccggguuaacauucauccg....................	133	0	seq
........................................................................caccggguuaacauuGauccg....................	1	1	seq
........................................................................caccggguuaacauuAauccg....................	8	1	seq
........................................................................caccggguuaacauucauccgU...................	1	1	seq
.........................................................................accggguuaacauucaucc.....................	1	0	seq
.........................................................................accggguuaacauucauccg....................	27	0	seq
.........................................................................Uccggguuaacauucauccg....................	1	1	seq
.........................................................................Gccggguuaacauucauccg....................	2	1	seq
.........................................................................aAcggguuaacauucauccg....................	1	1	seq
..........................................................................ccggguuaacauucaucc.....................	1	0	seq
..........................................................................ccggguuaacauucauccg....................	12	0	seq
...........................................................................cggguuaacauucauccg....................	8	0	seq
............................................................................ggguuaacauucauccgU...................	1	1	seq
```
